# General Practice Statistics in Australia: Pushing a Round Peg into a Square Hole

**DOI:** 10.3390/ijerph19041912

**Published:** 2022-02-09

**Authors:** Julie Gordon, Helena Britt, Graeme C. Miller, Joan Henderson, Anthony Scott, Christopher Harrison

**Affiliations:** 1 WHO Collaborating Centre for Strengthening Rehabilitation Capacity in Health Systems, University of Sydney, Sydney, NSW 2006, Australia; 2 Sydney School of Public Health, University of Sydney, Sydney, NSW 2006, Australia; helena.britt@sydney.edu.au (H.B.); graeme.miller@sydney.edu.au (G.C.M.); joan.henderson@sydney.edu.au (J.H.); 3 Melbourne Institute of Applied Economic and Social Research, University of Melbourne, Melbourne, VIC 3053, Australia; a.scott@unimelb.edu.au; 4 Menzies Centre for Health Policy and Economics, Sydney School of Public Health, University of Sydney, Sydney, NSW 2006, Australia; christopher.harrison@sydney.edu.au

**Keywords:** general practice, health services research, primary health care

## Abstract

In Australia, general practice forms a core part of the health system, with general practitioners (GPs) having a gatekeeper role for patients to receive care from other health services. GPs manage the care of patients across their lifespan and have roles in preventive health care, chronic condition management, multimorbidity and population health. Most people in Australia see a GP once in any given year. Draft reforms have been released by the Australian Government that may change the model of general practice currently implemented in Australia. In order to quantify the impact and effectiveness of any implemented reforms in the future, reliable and valid data about general practice clinical activity over time, will be needed. In this context, this commentary outlines the historical and current approaches used to obtain general practice statistics in Australia and highlights the benefits and limitations of these approaches. The role of data generated from GP electronic health record extractions is discussed. A methodology to generate high quality statistics from Australian general practice in the future is presented.

## 1. Introduction

General practice is the foundation of the Australian healthcare system, as general practitioners (GPs) are the gatekeepers for patient access to many other health services. Reliable data about GP clinical activity is needed for statistical analysis by primary care and public health researchers, those involved with health policy, health services planning and costing, GP educators, health consumers, and those involved in the development and production of health treatments and interventions. In this commentary article we will discuss the historical and current approaches used to obtain statistics in Australian general practice, highlight benefits and limitations in these approaches, and outline a proposed methodology to generate high quality statistics from general practice in the future.

## 2. Background

General practice forms a core part of the Australian healthcare system, often representing a patient’s initial contact with the system. GPs in Australia manage patients across their lifespan, manage chronic health conditions and multimorbidity, and provide preventive healthcare. They also have a ‘gatekeeper’ role, providing referrals for patients to access other services including care from non-GP specialists, and subsidized care from allied health professionals for patients with chronic conditions. Currently, patients are free to attend one or more GPs of their choice, and are not assigned to a particular GP or practice. While patients have this freedom, most attend the same practice for continuity of care ([1], Chapter 15). In Australia, general practices are usually private medical practices providing “comprehensive, coordinated and continuing medical care drawing on biomedical, psychological, social and environmental understandings of health” [2].

In 2019, there were over 37,000 GPs in Australia, working across 8147 general practices [3].

According to data from the World Bank, 86% of the Australian population lived in urban areas in 2020 [4], primarily along the East Coast. Accordingly, in 2019, approximately three-quarters (74.5%) of full-time equivalent GPs reported working in major cities [3]. In any one year, approximately 87% of the population see a GP, and on average, there were six GP visits per head of population in Australia in 2015–2016 [1].

In March 2021, Australia had a population of 25.7 million people [5]. Funding of health services in Australia is the responsibility of the federal (national) and state/territory (regional) governments. Spending on health totaled $197.7 billion (Australian) dollars in 2018–2019, equating to $7772 per head of population [6]. Health spending represented 10% of gross domestic product.

In 2018–2019 $65.5 billion was spent on primary health care [6], which incorporated general practice, allied and community health, and pharmacy (excluding Indigenous health care). General practice is primarily funded by the federal government on a ‘fee for service’ model, where GPs can charge any fee they wish, and patients receive a fixed subsidy according to the Medicare Benefits Schedule (MBS), a catalogue of medical services for which a rebate can be claimed from the government [7]. If the fee for a consultation or service provided is equal to the Medicare subsidy, then the consultation is ‘bulk billed’. Around 87% of GP services are bulk-billed [3]. If not, then the patient pays an out-of-pocket cost decided by the GP. For patients with very high out-of-pocket costs for GP and non-GP specialist consultations, additional subsidies are provided through the Medicare Safety Net [8]. Medicare items for GP consultations are based on broad estimates of consultation length and complexity. Limited items are related to specific diseases or for specific population groups (e.g., annual health assessments for patients aged 75+ years, or chronic disease management plans for patients with diabetes). Other Medicare-rebatable services include pathology tests, imaging tests and procedures undertaken. A separate Pharmaceutical Benefits Scheme (PBS) provides public subsidies for most prescribed medications dispensed by pharmacists [9].

The important role of general practice within the wider healthcare system has been recognized for some time. White et al. introduced a framework in Britain in 1961 to depict the organization of health care, demonstrating that within a population of 1000 adults, 250 (or 25%) will consult a physician (i.e., a primary care doctor or GP) in any one month. Nine of these 250 patients seeking care will be hospitalized, and five referred to another physician for care [10]. The overall stability of this framework has been established over time [11,12]. The aim of generating statistics from general practice is therefore not only to understand clinical activity undertaken in this setting, but to understand the health of the population overall.

In August 2019, the Australian Government released ‘Australia’s long-term national health plan’. The plan contained four ‘pillars’ (focus areas), the first of which was to strengthen the role of primary health care in the Australian healthcare system [13]. Later that year, a Primary Healh Reform Steering Group was established, focusing on the development of a ten-year plan for primary health care [14]. The draft report for the ‘Primary Health Care 10 Year Plan’ was released in October 2021 for consultation. The draft reforms are wide-ranging, containing changes to the funding models used in general practice, methods of general practice care delivery, and the introduction of patient registration at a single GP practice. The need for data to guide policy and quality improvement is reinforced in the plan [15].

If the draft reforms are implemented, there will likely be a multitude of changes to the current model of general practice in Australia. The proposed introduction of patient registration at a GP practice might further the role of the GP as central to population health. High-quality evidence-based statistics are required, to establish a baseline dataset for current general practice care delivery, and to assess the impact and effectiveness of any implemented reforms. This presents a timely opportunity to review the current state of general practice statistics in Australia.

## 3. History of General Practice Statistics in Australia

A detailed history of general practice data collection and analysis in Australia has been described elsewhere [16]. The first general practice survey was conducted by Dr Clifford Jungfer (GP) and Dr John Last (epidemiologist) in 1959–1960, with support from the (then) Australian College of General Practitioners [17]. This was followed by a National Morbidity Survey in 1962 [18]. Meanwhile, Dr Kevin Cullen, a GP in the town of Busselton, Western Australia, began the Busselton Health Study, a longitudinal study of population groups within Busselton conducted between 1966 and 1981. The Busselton Health Study was based on repeated cross-sectional surveys comprising questionnaires and blood tests to investigate the health of the study population, and identify health indicators that predicted future disease [19].

The Australian General Practice Morbidity and Prescribing Survey was conducted from 1969 to 1974, started by the Royal Australian College of General Practitioners’ research committee, and led by Dr Charles Bridges-Webb [20]. The methods used in this study became the foundation for subsequent surveys of general practice clinical activity, including the Australian Morbidity and Treatment Survey (1990–1991) [21] and the Bettering the Evaluation and Care of Health (BEACH) study (1998–2016) [1].

For 18 years, the BEACH study described the clinical activity undertaken by GPs in Australia [1]. In BEACH, 1000 randomly selected GPs were sampled in each year of the study. Each GP participant recorded de-identified data for about 100 consecutive patient encounters on structured paper forms. Data collected included some patient demographics (e.g., date of birth, patient sex, postcode, Indigeneity), the patients’ reasons for encounter (up to three), problems managed at the encounter (up to 4), medications prescribed/supplied/advised for purchase, for each problem, other treatments provided for each problem (including procedures and clinical treatments, such as advice and counselling), and pathology and imaging requests for each problem. Importantly, each management action was explicitly linked to the problem for which that action was taken. More detailed methods for the BEACH study can be found elsewhere [1]. BEACH closed in 2016 due to the withdrawal of support from the federal government (both funding and loss of the random samples of GPs provided) and wider losses of research support from industry partners [16]. With a final database spanning 18 years and approximately 1.8 million GP–patient encounter records, BEACH data were used to investigate the problems managed by GPs, how GPs managed these problems during consultations, and how the quality of care provided by GPs compared to evidence-based guidelines. BEACH data also identified changes in general practice clinical activity over time [22] and provided evidence about numerous policy areas, including time spent on patient care not able to be claimed through the MBS [23], the potential cost of freezing MBS item rebates [24] and (using length of consultation data) disproved statements that GPs were providing so-called ‘six minute medicine’ [25]. It was widely recognized that the closure of BEACH created a gap in data available about general practice [26]. Irving et al., in their investigation of primary care physician consultation time, presented a rather thorough international comparison of general practice data collection methods through their systematic review of 67 countries, and concluded that the Australian BEACH study “represents the gold standard for consistent reporting” [27].

The end of BEACH coincided with the closure of a number of other sources of data about general practice in Australia. Government funding was withdrawn from the Australian Primary Health Care Research Institute in 2015 [28]. The Medicine in Australia: Balancing Employment and Life (MABEL) study, a longitudinal study about the medical workforce, ended in 2019, after 11 waves of data collection. This study provided numerous insights on access to medical care from between 3000 and 4000 GPs, followed up each year, including the drivers of hours worked, job satisfaction, and factors influencing recruitment and retention in rural areas [29]. The Australian Government’s Medical Research Future Fund, established to provide grants for health and medical research, is reported to allocate less than 1% of total funding to primary care research [30]. Currently, many gaps exist in the statistics available from general practice, both in terms of the data collected and the research conducted [31].

## 4. Current Status of General Practice Clinical Activity Data

Limited administrative data are available about general practice from the MBS and the PBS. The MBS has records of the consultation items claimed by GPs from Government, but these provide very limited understanding of the clinical content of the consultation or the characteristics of the GPs. Similarly, the PBS contains data about subsidized medications dispensed by pharmacies, but does not include data about the clinical indication (i.e., symptom or diagnosis) for which the medication was prescribed. To obtain data about the clinical content of GP consultations, we need to look elsewhere.

General practice was one of the early adopters of computerized clinical records, with government incentives to use computers available as early as 1998 [32]. Computerization began in the early 1990s, and some of the early systems developed (e.g., Medical Director) are still commonly used today [33]. There are now at least eight brands of electronic health records (EHRs) currently used in Australian general practice [33]. According to BEACH data, in 2014–2015, 97.5 % of Australian GPs reported that they used a computer for one or more purposes. However, only 70.7% used paperless medical records while 25.5% used hybrid (paper and electronic) records [22]. The MABEL survey in 2018 also asked about GPs’ use of digital technology for a range of tasks, and found (for example) almost 90% of GPs using digital technology to view imaging pathology and results [34]. These data demonstrate that while GPs have a high uptake of computerized medical records and digital technologies, some still rely on paper for some activities.

While the BEACH study was conducted on paper, some GPs said they would have preferred to be able to download data from their practice electronic health records (EHRs) to be used in the study. There were two primary reasons that structured paper forms were used in BEACH. First, to facilitate the linkages between the problems managed and all management actions provided for each problem. The problem–management linkage in BEACH ensured the GP specifically linked the prescription of a medication to the problem for which it was prescribed. It remains extremely difficult, if not impossible, to obtain these linkages from EHR data. This has led some researchers, using GP EHR data, to secondarily link each medication to a problem in the record on the basis of ‘probability’. However, medications will often have multiple possible indications, let alone other off-label uses, making it difficult to know what health problem it is treating, and making matching by assumption highly unreliable. Second, BEACH was a study of GP clinical activity. The structured paper forms were inherently transportable, so that GPs who worked in multiple practices could take the forms between practices, or to home visits or nursing home visits. Secondary data entry by trained clinical coders, while time consuming and costly, facilitated consistent coding of the data to improve data quality.

In the absence of BEACH data since 2016, statistics from general practice have become focused on data extracted from EHRs. There are numerous research programs in Australia that rely on de-identified data extracted from GP EHRs, including:

(1) MedicineInsight (NPS MedicineWise);
(2) Data for Decisions (University of Melbourne);
(3) Primary Health Insights (led by WA Primary Health Alliance).

Data extraction from EHRs may be as basic as a simple export tool. More complex extraction tools have been developed specifically for this purpose [35], for example GRHANITE (University of Melbourne) [36], the CAT4 tool (Pen Computing) [37] and POLAR GP [38]. These tools can be used at multiple levels—for clinical audit or quality improvement activities at the practice, or by the local health region (called Primary Health Networks or PHNs in Australia), or to provide data to research programs at a wider level.

## 5. The Use of EHR Data for Research and Statistics

The automated extraction of data already collected during the clinical patient encounter creates a database of ‘passive’ data that can be used for statistics and research. While the primary purpose of data collection in an EHR is for patient care, making these data available for research and statistics minimizes the effort for individual GPs (who are often poor in time [39,40]) to participate in studies for multiple research groups. However, organizing and performing data extraction does involve time and effort for the practice. GPs report that it is often practice staff who undertake these activities [40], so the process is not entirely automated and does have a cost, although this is not always perceived as a barrier [41].

Passively collected data creates large volumes of data that can be interrogated in many ways. This provides greater scope to examine the management of rare phenomena. Theoretically, for patients who regularly attend the same practice, EHR data extraction allows for the longitudinal analysis of a patient’s journey over time, providing the potential to assess medical interventions and long-term health outcomes. This is limited though, if patients attend multiple practices (e.g., while travelling or for convenience) or change practices for any reason, resulting in incomplete data.

### 5.1. Variability in EHR Design

Interoperability of data requires standard approaches to data design structures, data field names and their associated definitions, and the coding and classification of relevant data fields. Standardization is required to enable data to be combined from different EHRs for clinical audits and research, and to facilitate the transfer of patient care between different healthcare providers (e.g., referrals). All of the GP EHRs used in Australia have been developed independently, which limits such interoperability and the ability to generate meaningful data from general practice EHRs, both for clinical and statistical purposes [33,35].

There are differences in the underlying designs of the EHR database structures, including the data field names, their definitions, and how data fields are or are not linked. There are also vast inconsistencies in the use of clinical classifications and terminologies, including the type of clinical terminology used (e.g., termsets developed by individual EHR developers, ICPC-2 PLUS [42] or SNOMED CT-AU [43]). In most EHRs, clinicians can choose whether to enter a term from one of these termsets or to enter free text [33]. As a result, most EHR research databases extract data from only some of the available EHRs, limiting the representativeness of the data. For example, MedicineInsight extracts data from the two most commonly used EHRs [44], each of which uses a different coding system.

### 5.2. Data Completeness

The quality of research and statistics is only ever as good as the quality of the data contained in the record from which the data are extracted. Data accuracy in EHRs has been found to be variable [35,41], which is likely to impact on research quality. In one recent Australian study, approximately 13% of probable cases did not have a coded diagnosis, and were identified through the presence of one or more other diabetes management indicators [45].

Bailie et al. (2015) identified difficulties in calculating denominators in patient data extracted from EHRs. Numerous reasons were given, including incomplete data entry, differing requirements and compatibility between EHRs and data extraction tools, and differences in the definition used for active or regular patients. The authors concluded that the inconsistencies identified limited the usefulness and reliability of the EHR data [46].

### 5.3. The Medical Record as an ‘Aide Memoir’

The primary purpose of the EHR is to capture data that relates to the clinical care of the patient, not to obtain data for research purposes [47]. Henderson et al. (2019) suggest that time-poor GPs may only enter the data they regard as important for patient care, which may not always reflect the data that are important for research. This limits the capability of using EHR data for research purposes [45].

The medical record has long been regarded as an ‘aide memoir’, or memory aid, rather than as a complete record of the patient’s care. Even with the advent of EHRs, this association has continued. In a benchmarking study that examined the prevalence of diabetes using BEACH data and extracted data from one Australian EHR, the prevalence of diabetes was lower when using the extracted EHR data from the ‘diagnosis’ data element. However, the authors found that they could obtain a comparable prevalence estimate by identifying proxies that indicate the presence of diabetes (e.g., free text searches for diabetes in other parts of the record, medications used to treat diabetes, use of MBS item numbers only used in relation to diabetes). Importantly, the authors noted that this approach would be less reliable for other clinical conditions where proxy measures may not work [45]. Interestingly, MedicineInsight does not extract free text data, as it may contain identifiable information that could compromise privacy [44].

### 5.4. Privacy and Information Protection

The extraction of data from EHRs for statistical and research purposes usually involves the transfer of the exported patient data to a third party (e.g., government department or University researcher). Concerns have arisen in Australia about patient privacy and information protection [35,40,41]. The removal of information from extracted data that would identify a patient has been highlighted as being of primary importance to researchers [35,41,48], GPs [40,41] and other practice staff [41]. The need for independent governance oversight of programs that involve extracted EHR data has also been emphasized [35,48].

At present, most data extraction from general practice EHRs involves the whole of practice data, where data are extracted about all patient encounters [44]. Concerns may arise if individual GPs within a practice are not willing to have data about their clinical activity included in a download, or when patients do not give permission for their data to be downloaded.

## 6. A Fresh Approach

We propose a new approach to improve the production of high-quality data about general practice clinical activity. This proposal is based on the following principles:

(1) Data from general practice can provide an excellent overview of the health of the population overall;
(2) Using the GP as an ‘expert interviewer’ to curate data can facilitate data with higher levels of accuracy than patient self-report;
(3) It is not necessary to collect data about all the patients, all the time. The BEACH study demonstrated that the production of structured data, about a sample of patients, can generate high-quality statistics from general practice for use in policy planning, education, and research;
(4) The sample of patients must be representative of the patient population to ensure validity and reliability;
(5) Data need to be longitudinal for the investigation of outcomes of care, including care provided by other health services (e.g., specialists, hospitals);
(6) The capacity to review the patient’s experience with the health system overall, through linking general practice data to that from other health agencies, is encouraged.

Building on the structure of the BEACH interface for active data collection, we propose developing a hybrid active + passive data collection based on data extraction from EHRs with subsequent data curation from GPs to review the quality of extracted data and complete gaps in the dataset. A specialized data extraction tool would be required to extract relevant data from the GP EHR. To circumvent problems experienced with current EHR data extractions, the GP would curate the data for completeness and validity.

We propose that two data templates are required:

(1) A health summary template where the GP extracts a health summary from the EHR (similar to the patient summary currently contained in the EHR), followed by a ‘check and curate’ process, in which the GP reviews the accuracy and completeness of the data extracted. For example, is the patient’s problem list accurate? Are medications listed that the patient no longer takes, or are there over-the-counter medications taken regularly that should be added? There are also additional data elements not currently included in GP EHRs that could be captured in this process. For example, capture of data about social determinants of health (e.g., education level, household income) would contribute to a greater understanding of a patient’s health and related health outcomes;
(2) An encounter summary template where the GP extracts and curates data about an individual GP–patient encounter. This data extraction would be based on data elements that were collected in BEACH using a problem-oriented structure. The GP would curate the data by completing areas within the template that are missing and add linkages between problems managed and their treatments.

For each of these, minimum datasets based on a problem-oriented record structure with in-built coding and classification systems would be required for the purposes of data extraction, encryption and transfer to researchers, and subsequent data analysis.

Initially, these could be used to provide cross-sectional data from a representative sample of patients who attend general practice. A second stage of research would involve use of the tool as the basis for longitudinal data collection, whereby a sample of patients are recruited to the study and their data are extracted and curated at every visit. The addition of data about other health services received between GP visits (e.g., specialist, hospital or allied health visits), added and curated by the GP, would enhance knowledge about patients’ broader experiences with the health system.

The strength of this approach is the focus placed on the importance of record structures, data linkages, coding and classification systems, and in the general application of standards required for the success of the model.

This approach will improve the understanding of morbidity and management within the general practice population and provide baseline data for further research and evaluation examining interventions to improve quality of care for general practice patients. It has some utility for use in GP clinical audits and quality assurance.

## 7. Conclusions

The Primary Health Care Reforms currently under consideration reference the ‘quadruple aim’ of health care, improving: (1) people’s experiences with health care; (2) population health; (3) cost-efficiency of the health system; and (4) work life for healthcare workers [49]. The first three of these are quantifiable measures that rely on the availability of relevant data, and statistical analysis of these data, to assess the effectiveness of any reforms implemented to achieve these aims.

There is a reliance on data currently contained in GP EHRs to answer these questions, as shown in the reform policy and in initiatives such as the Australian Institute of Health and Welfare’s Primary Health Care Data Asset. Current forms of data extraction from EHRs might be economically preferable and can answer some questions, but they cannot answer all of them. The temptation to use these datasets may equate to ‘trying to fit a square peg into a round hole’, an idiom that implies a solution that is unfit for purpose. Rather than accepting or ignoring the limitations of EHR data that currently exist, why not be aspirational? How can we achieve better statistics from general practice that are able to inform both the patient and provider experience, and can be used for system planning?

COVID-19 has changed the way general practice services are conducted in Australia. The availability [50] and use [51] of telehealth services represents a dramatic shift in the way general practice services are provided to the public. However, there are little data available about how COVID-19 has changed the clinical activity undertaken by GPs and the quality of care provided through telehealth. Changes to the GP workforce resulting from COVID-19, and the future intentions of the GP workforce may have also been impacted by the pandemic, but with little data available it is impossible to quantify these. The approach presented in this paper for improving clinical activity data should be complemented by reinvestment in longitudinal data about the GP workforce, lost by the cessation of the MABEL study.

The approach to general practice data outlined in this paper may not answer every question that could be asked about general practice, but it would go a long way in overcoming the current deficiencies, and would produce national, valid, reliable statistics from Australian general practice.

## Data Availability

Not applicable.
